# Comparative Efficacy of First and Second Generation long-acting injectable antipsychotic upon schizophrenic patients: a systematic review and network metaanalysis.

**DOI:** 10.1192/j.eurpsy.2023.2301

**Published:** 2023-07-19

**Authors:** R. Medrano, E. Saucedo, C. Mancias, C. Saucedo

**Affiliations:** 1 Psychiatry, Departamento de Psiquiatria, Hospital Universitario “Dr. Jose Eleuterio Gonzalez”; 2 Psychiatry, Centro de Neurociencias avanzadas, UANL, Monterrey, Mexico

## Abstract

**Introduction:**

Long-acting injectable antipsychotics (LAIAs) are currently the most effective alternative for patients with schizophrenia who exhibit poor adherence. LAIAs can lead the course of treatment with the potential to increase adherence in schizophrenia treatment.

**Objectives:**

Present the results of a network metaanalysis on the comparative efficacy of LAIs in schizophrenia.

**Methods:**

Included trials of adults with schizophrenia compared the efficacy of LAI vs LAI or placebo through the Positive and Negative Syndrome Scale (PANSS). Efficacy was evaluated through the standarized mean differences (SMD) from baseline to endpoint in the PANSS total scores.

**Results:**

Results from 15 studies reported usable results for PANSS score (five antipsychotics compared) are shown in Figure 1. In hierarchical order, haloperidol, aripiprazole, risperidone, and paliperidone reduced the PANSS score significantly more than other drugs.

**Image:**

**Figure:**
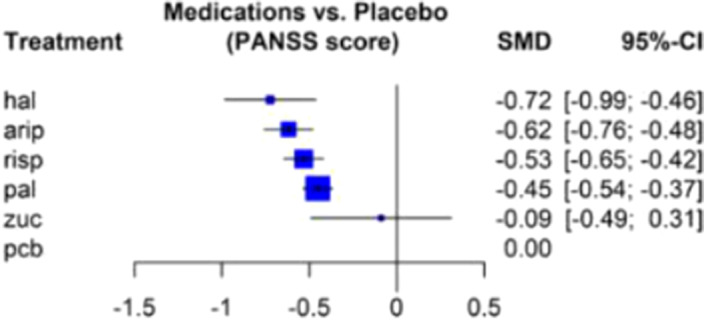

**Conclusions:**

Most LAIAs are equally efficient at reducing overall symptoms, and differences between individual LAIAs are non-significant.

**Disclosure of Interest:**

None Declared

